# Veno-venous ECMO as a platform to evaluate lung lavage and surfactant replacement therapy in an animal model of severe ARDS

**DOI:** 10.1186/s40635-020-00352-w

**Published:** 2020-10-27

**Authors:** Robert Qaqish, Yui Watanabe, Marcos Galasso, Cara Summers, A adil Ali, Mamoru Takahashi, Anajara Gazzalle, Mingyao Liu, Shaf Keshavjee, Marcelo Cypel, Lorenzo Del Sorbo

**Affiliations:** 1 Latner Thoracic Surgery Research Laboratories, Toronto, Canada; 2 Interdepartmental Division of Critical Care Medicine, University Health Network, Toronto General Hospital, 585 University Avenue, PMB 11-122, Toronto, ON M5G 2N2 Canada; 3 University of Toronto, Toronto, ON Canada

**Keywords:** Animal model, Extracorporeal membrane oxygenation, Inflammatory mediators

## Abstract

**Background:**

There are limited therapeutic options directed at the underlying pathological processes in acute respiratory distress syndrome (ARDS). Experimental therapeutic strategies have targeted the protective systems that become deranged in ARDS such as surfactant. Although results of surfactant replacement therapy (SRT) in ARDS have been mixed, questions remain incompletely answered regarding timing and dosing strategies of surfactant. Furthermore, there are only few truly clinically relevant ARDS models in the literature. The primary aim of our study was to create a clinically relevant, reproducible model of severe ARDS requiring extracorporeal membrane oxygenation (ECMO). Secondly, we sought to use this model as a platform to evaluate a bronchoscopic intervention that involved saline lavage and SRT.

**Methods:**

Yorkshire pigs were tracheostomized and cannulated for veno-venous ECMO support, then subsequently given lung injury using gastric juice via bronchoscopy. Animals were randomized post-injury to either receive bronchoscopic saline lavage combined with SRT and recruitment maneuvers (treatment, *n* = 5) or recruitment maneuvers alone (control, *n* = 5) during ECMO.

**Results:**

PaO_2_/FiO_2_ after aspiration injury was 62.6 ± 8 mmHg and 60.9 ± 9.6 mmHg in the control and treatment group, respectively (*p* = 0.95) satisfying criteria for severe ARDS. ECMO reversed the severe hypoxemia. After treatment with saline lavage and SRT during ECMO, lung physiologic and hemodynamic parameters were not significantly different between treatment and controls.

**Conclusions:**

A clinically relevant severe ARDS pig model requiring ECMO was established. Bronchoscopic saline lavage and SRT during ECMO did not provide a significant physiologic benefit compared to controls.

## Background

Acute respiratory distress syndrome (ARDS) is an important public health problem with an incidence of 78.9 per 100,000 person-years and an associated mortality rate between 27–45% [1, 2]. Despite decades of research, there are limited therapeutic options directed at the underlying pathological processes [3, 4], and supportive care with mechanical ventilation remains the cornerstone of management [5]. When conventional strategies fail to provide adequate support, patients are treated with an escalation of interventions, including veno-venous extracorporeal membrane oxygenation (VV-ECMO), which is increasingly used worldwide for this indication [6, 7]. However, the overall management is limited by the lack of effective pharmacological therapies [3].

Experimental therapeutic strategies have focused on targeting lung surfactant as it becomes deranged in ARDS [8, 9]. Inflammatory mediators and enzymes are released within the alveolar unit and interfere with the production, recycling and architecture of surfactant, rendering it dysfunctional [8–11]. Surfactant therapy for neonates with respiratory distress syndrome is well established, however the results of clinical trials investigating the use of surfactant in adults with ARDS have at best been discouraging [8–11]. The most recent human trial that evaluated intratracheal instillation of surfactant in moderate/severe ARDS patients failed to show improvements in oxygenation or mortality [12]. Explanations surrounding the lack of efficacy of surfactant replacement therapy (SRT) in adults may involve poor alveolar delivery secondary to insufficient dosing and poor efficiency of administration [11]. Findings based on fluid mechanical computational modeling of SRT have suggested that higher volumes of surfactant and bronchoscopic administration may improve the homogeneity and efficiency of distribution [11]. Indeed, bronchoscopic administration of surfactant in human and large animal studies has shown promise in ARDS and transplant clinical models [13–18]. Moreover, a recent study evaluated the therapeutic effects of saline lavage and exogenous SRT in an experimental model of aspiration-induced acute lung injury [16]. In this model, ex vivo lung perfusion (EVLP) was used as a platform to allow large-volume lavage followed by SRT. During EVLP, the combination of saline lavage followed by SRT resulted in better physiologic lung function and reduced inflammation compared to controls. These results suggest that lung lavage may act to remove the cause of lung injury, including aspiration contents, inflammatory mediators, and aspiration-induced dysfunctional surfactant, which can then be replaced with exogenous surfactant.

However, these findings may not be easily translated into clinical practice, since severe hypoxemia may prevent the safe delivery of adequate amounts of lavage fluid and exogenous surfactant. Moreover, EVLP is currently not a therapeutic option beyond lung transplantation. Nonetheless, the efficacy of this innovative therapeutic approach can be studied in an experimental model of severe ARDS supported by VV-ECMO. Since VV-ECMO efficiently provides adequate gas exchange with either minimal or no contribution of injured lungs, it allows the safe administration of bronchoscopy-based treatments during mechanical ventilation even in severe ARDS, including large-volume lung lavage followed by SRT.

Therefore, we sought to evaluate the in vivo effect of large-volume saline lung lavage followed by SRT in a pre-clinical aspiration-induced model of severe ARDS.

## Methods

### Animal preparation

Animals in our study were treated in accordance with the ‘Guide for the Care and use of Laboratory Animals’ (National Research Council). The Toronto General Research Institute approved our protocol. Detailed methods can be found in the online data supplement.

**Fig. 1:**
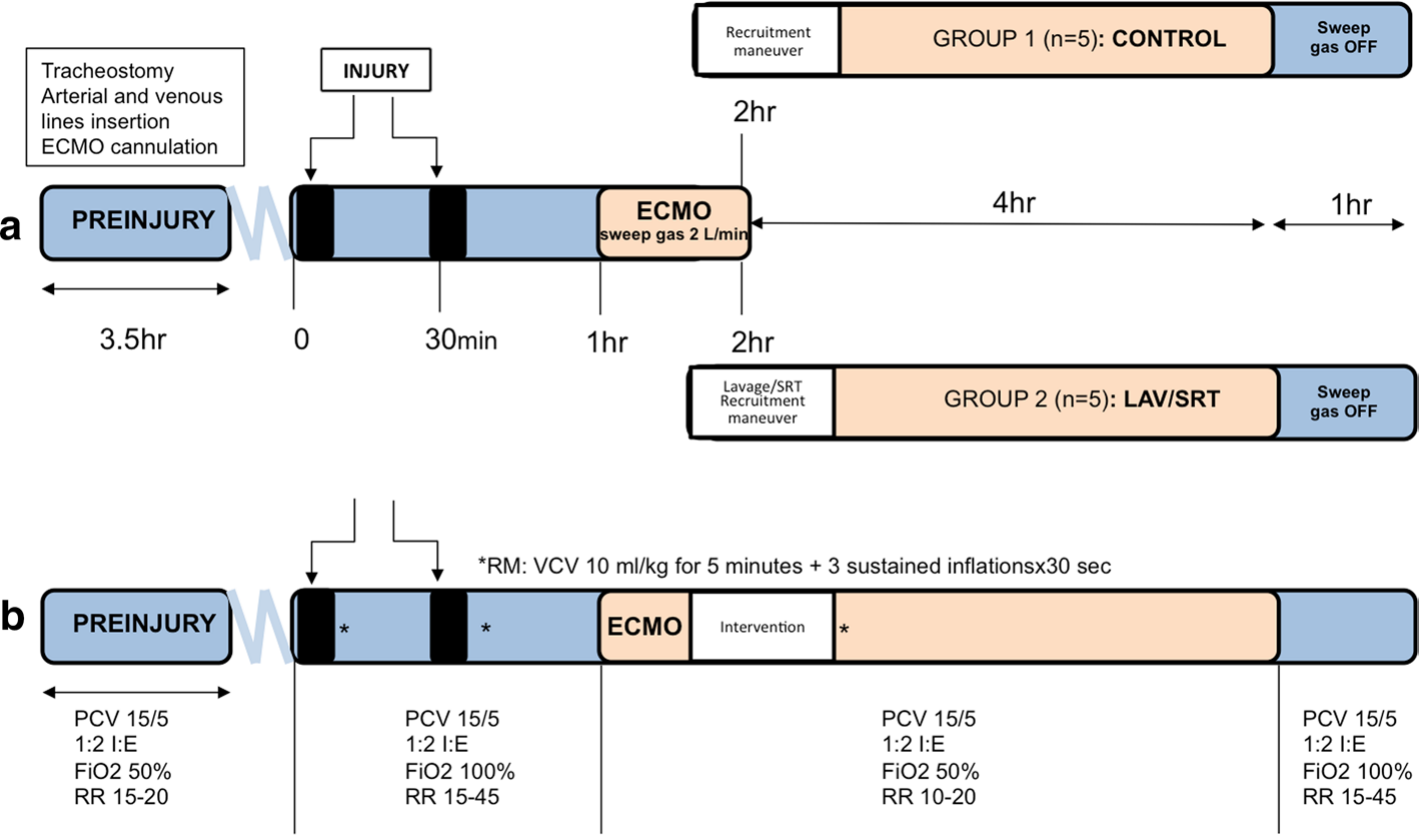
**a** Experimental outline and time course and **b** mechanical ventilation strategy during ‘PREINJURY’, ‘INJURY’, ‘ECMO’ and ‘OFF’ ECMO phases of the experiment

Yorkshire male domestic pigs (29–37 kg) were anesthetized, tracheotomized and mechanically ventilated. Carotid arterial line and pulmonary artery (PA) catheter were inserted for hemodynamic monitoring. Figure 1 shows the experimental outline.

### ECMO management

Before lung injury, under systemic heparinization open jugular and femoral ECMO cannulation was performed. At 1 h post-injury (ARDS induction), ECMO sweep gas was increased from 0 to 2 L/min and target oxygen saturation was maintained above 88% with MAP > 65 mmHg.

### ARDS induction

Two bronchoscopic instillations of gastric juice (GJ) (4 mL/kg and 2 mL/kg, pH 1.6) were administered 30 min apart to target a PaO_2_/FiO_2_ ≤ 100 mmHg [16, 19]. Two hours post-injury, blood and bronchoalveolar lavage fluid (BAL) were collected for future analysis and CXR was performed.

### Randomization

Ten animals were randomized post-injury to receive during ECMO either treatment with lavage (LAV) + surfactant (SRT) + recruitment maneuver (RM), or RM alone (controls). RM was performed with 3 sustained inflations, 10 s each at airway pressure of 30 cmH_2_O during a 5-min period of mechanical ventilation with 10 mL/kg of tidal volume.

### Treatment group (LAV/SRT, *n* = 5)

2 h post-injury, 200 mL (10 mL × 20 segments) of saline were used to lavage the lungs and promptly recovered via bronchoscopy. Surfactant (135 mg/kg, BLES® Biochemicals Inc. SP-B/C, London ON) was administered via bronchoscopy after the lavage. During surfactant instillation and for 5 min following, a recruitment maneuver was performed to facilitate surfactant distribution.

### Control group (controls, n = 5)

Two hours post-injury the animals received a recruitment maneuver.

### Post-treatment time course

After the intervention, animals were supported on VV-ECMO and monitored for 4 h. Just prior to withdrawal of ECMO support, BAL and blood samples were collected for further analysis. The animals were monitored for 1 additional hour off ECMO (sweep gas 0 L/min) to evaluate the response to worsen gas exchange conditions and thus to enhance the potential subtle difference between the two experimental groups. At the end of the experiment, animals were euthanized and median sternotomy was performed for tissue collection.

### Cytokines

Cytokines in BAL, plasma and tissue homogenates were analyzed blindly (Millipore Sigma, Etobicoke, ON).

### Total bile acid (BA)

Total BA concentration was blindly measured from BAL taken pre-injury, 2 h post-injury and just prior to ECMO withdrawal (BQ Kits, San Diego CA).

### Statistical analysis

Data are presented as means ± standard deviation (SD). Groups of means were compared by Mann–Whitney and 2-way ANOVA for repeated measurements (GraphPad Prism 7.02, La Jolla, CA), as appropriate. Differences were considered statistically significant when the probability value was less than 0.05.

## Results

### ARDS induction

**Fig. 2:**
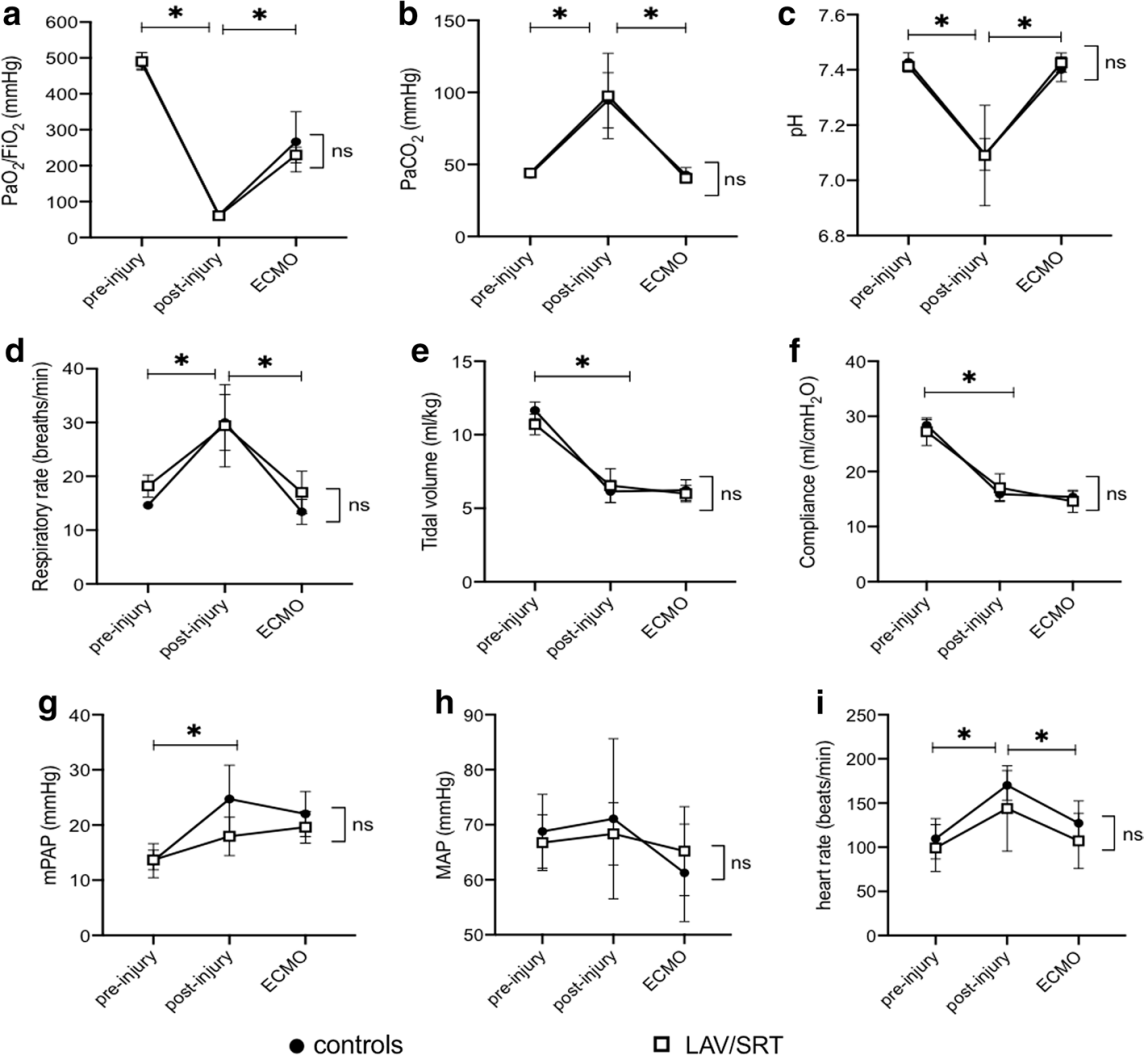
Physiologic variables before (pre-injury) and after instillation of gastric juice (post-injury), and following ECMO initiation. Data are presented as mean ± SD. **a** PaO_2_/FiO_2_; **b** PaCO_2_; **c** pH; **d** respiratory rate; **e** expired tidal volume in ml/kg; (F) compliance; **g** mean pulmonary artery pressure (mPAP); **h** mean arterial systemic pressure (MAP); **i** heart rate. Two-way ANOVA for repeated measures: *p < 0.05 pre-injury vs post-injury and post-injury vs ECMO

**Fig. 3:**
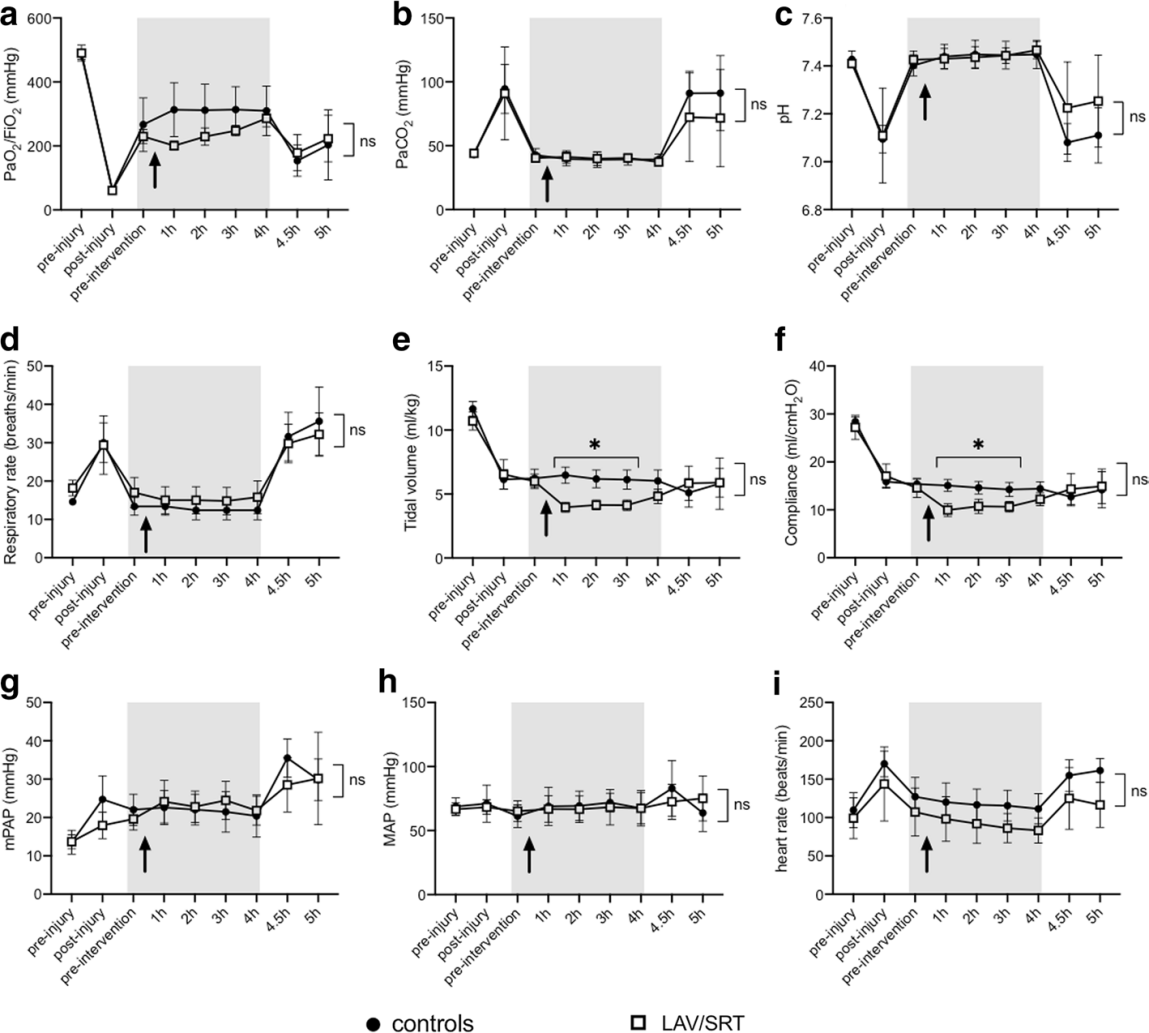
Lung physiology parameters. Data shown represent means ± SD. Gray-shaded area represents parameters evaluated on ECMO. Black arrow represents time at which intervention was performed. **a** PaO_2_/FiO_2_ (mmHg); **b** PaCO_2_; **c** pH; **d** respiratory rate; **e** expired tidal volume; **f** compliance; **g** mean pulmonary arterial pressure (mPAP); **h** mean systemic arterial pressure (MAP); **i** heart rate. Two-way ANOVA for repeated measures: *p < 0.05 controls vs LAV/SRT

The baseline and intraoperative variables of the animals in the two study groups were similar (Additional file 1: Table S1). The bronchoscopic instillations of GJ caused a significant decrease in oxygenation, which was comparable in both groups (*p* = 0.95) (Figs. 2a, e and 3). PaO_2_/FiO_2_ fell to 63 ± 8 (p < 0.01) and 61 ± 10 (*p* < 0.01) in the control and treatment group, respectively. Post-injury expired tidal volume, and hence respiratory system compliance, was also significantly reduced. Tidal volume dropped from 374 ± 6 mL (12 ± 1 mL/kg) to 197 ± 15 mL (6 ± 1 mL/kg) (*p* < 0.01) in controls and from 354 ± 33 mL (11 ± 1 mL/kg) to 215 ± 38 mL (6 ± 1 mL/kg) (*p* < 0.01) in treated animals (Fig. 2i). Moreover, CXR and bronchoscopy performed 2 h post-injury consistently showed bilateral infiltrates (Additional file 1: Figure S1 and S2, respectively). The injury also resulted in hemodynamic instability, with increased heart rate (HR) and pulmonary arterial pressure, which was comparable in the two experimental groups (Fig. 2). One animal required transient vasopressor support to maintain mean arterial pressure (MAP) > 65 mmHg during injury. The animals in the two groups received a similar amount of fluid during the experiment (Additional file 1: Table S1).

### VV-ECMO support

Gas exchange parameters stabilized in all the animals after the sweep gas was turned on to 2 L/min, despite a persistently low tidal volume and respiratory system compliance (Figs. 2, 3). The animals remained hemodynamically stable with a significant improvement in the HR. None of the animals suffered from ECMO-associated bleeding complications.

### Bronchoscopic saline lavage and surfactant

In animals randomized to the treatment group (*n* = 5) 200 mL of normal saline was bronchoscopically instilled in the airway and 134 ± 17 mL were recovered. The mean volume of surfactant instilled was 165 ± 9 mL. During treatment, the SpO_2_ remained > 88% in all cases. However, despite the performance of RM, in the intervention group the treatment caused an immediate drop in expired tidal volume (delta = 67 ± 14 mL) and PaO_2_/FiO_2_ (delta = 29 ± 35 mmHg), which subsequently improved (Fig. 3). PaO_2_/FiO_2_ ratio, expired tidal volume, respiratory rate and pCO_2_ remained stable in the control groups during the 4 h of ECMO support.

### VV-ECMO support withdrawal

**Fig. 4:**
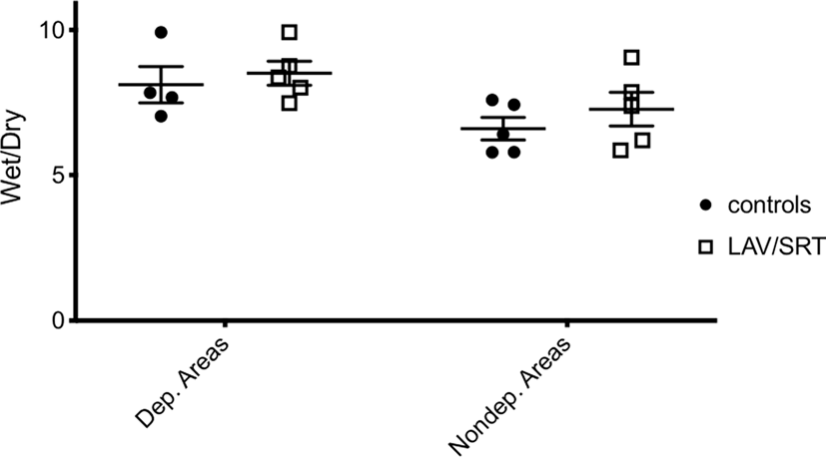
Wet-to-dry (W/D) lung weight ratios from dependent (dep.) lung and non-dependent (nondep.) lung tissue biopsies

After 4 h of ECMO support, the sweep gas was turned off and the animals were monitored for 1 final hour. In all cases, hemodynamic and physiologic parameters deteriorated similarly in the two groups (Fig. 3). At the end of the experiment, hemodynamic and respiratory variables were similar in the two groups. However, mean PaO_2_/FiO_2_ (223 ± 73 mmHg vs 203 ± 110 mmHg, *p* = 0.69), oxygen saturation (87 ± 18 vs 82 ± 9%, *p* = 0.19), pH (7.25 ± 0.2 vs 7.11 ± 0.1, *p* = 0.15) and heart rate (117 ± 29 vs 161 ± 16 beats/min, *p* = 0.06) showed a trend towards physiologic benefit of the treated animals compared to controls (Fig. 3). One animal (treatment group) required vasopressor support after ECMO support was removed (different from animal that required vasopressors during injury). The wet-to-dry ratio of the dependent (controls 6.6 ± 0.9 vs LAV/SRT 7.2 ± 1.3, *p* = 0.69) and non-dependent (controls 6.9 ± 2.8 vs LAV/SRT 7.9 ± 2.0, *p* = 0.31) lung zones were similar in both groups (Fig. 4).

### BA in BAL

**Fig. 5:**
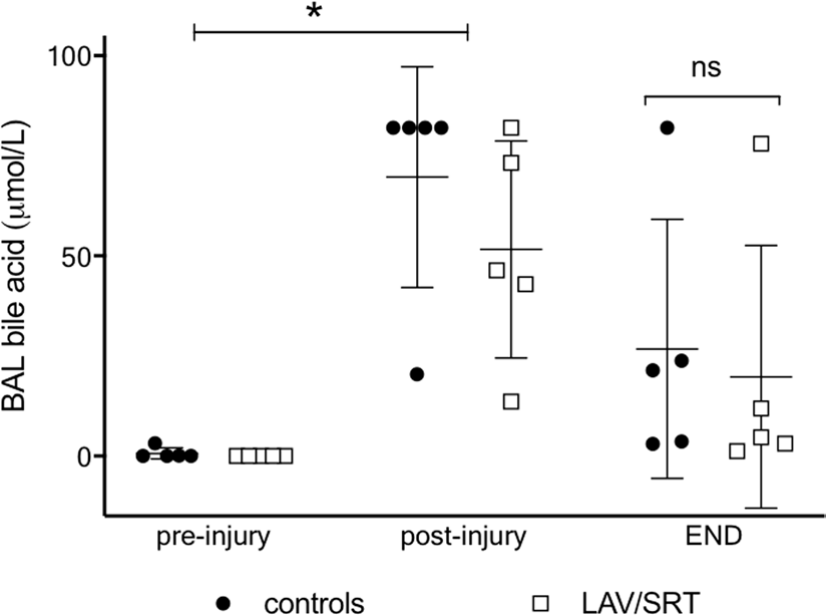
Total bile acid concentration (µmol/L) measured from bronchoalveolar lavage (BAL) taken prior to injury with gastric juice (pre-injury), 2 h after injury (post-injury) and at the end of the experimental protocol (controls or LAV/SRT). Two-way ANOVA for repeated measures: *p < 0.05 pre-injury vs post-injury. Mann–Whitney test: *p* > 0.05 controls vs LAV/SRT

Post-injury total BA levels were significantly elevated in comparison to pre-injury (< 0.02 μmol/L) concentrations and often surpassed the upper limit (82 μmol/L) of assay detection (Fig. 5). Prior to withdrawal of ECMO support, total BA levels were lower in both treatment (19.7 ± 33 μmol/L) and control (26.7 ± 32 μmol/L) groups compared to post-injury concentrations, but the treatment did not result in a statistically significant reduction in BA levels compared to controls (*p* = 0.55).

### Inflammatory cytokines

**Fig. 6:**
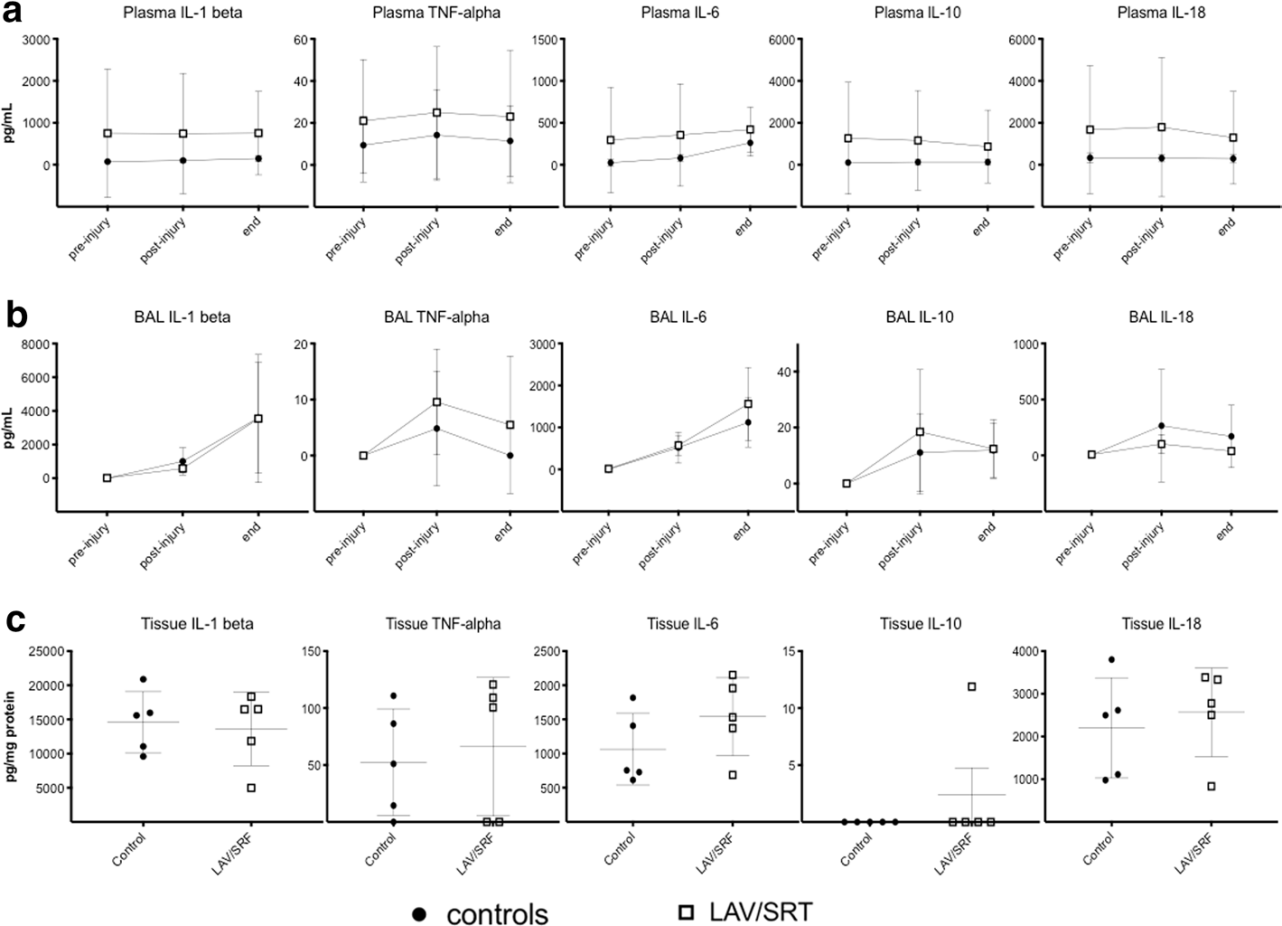
**a** Plasma and **b** bronchoalveolar lavage (BAL) cytokines profile over time (pre-injury, post-injury and end), and **c** cytokines from lung tissue biopsy taken at the end of the experiment. Data are presented as mean ± SD. Two-way ANOVA for repeated measures analysis in **a**, **b**: p > 0.05 controls vs LAV/SRT. Mann–Whitney test analysis in **c**, *p* > 0.05 controls vs LAV/SRT

No statistically significant difference in inflammatory cytokines concentration was found in plasma (Fig. 6a), BAL (Fig. 6b) or lung tissue (Fig. 6c) at the end of the experiment. Cytokines concentration over time was similar in the two experimental groups (Fig. 6).

### Histologic lung injury score

**Fig. 7:**
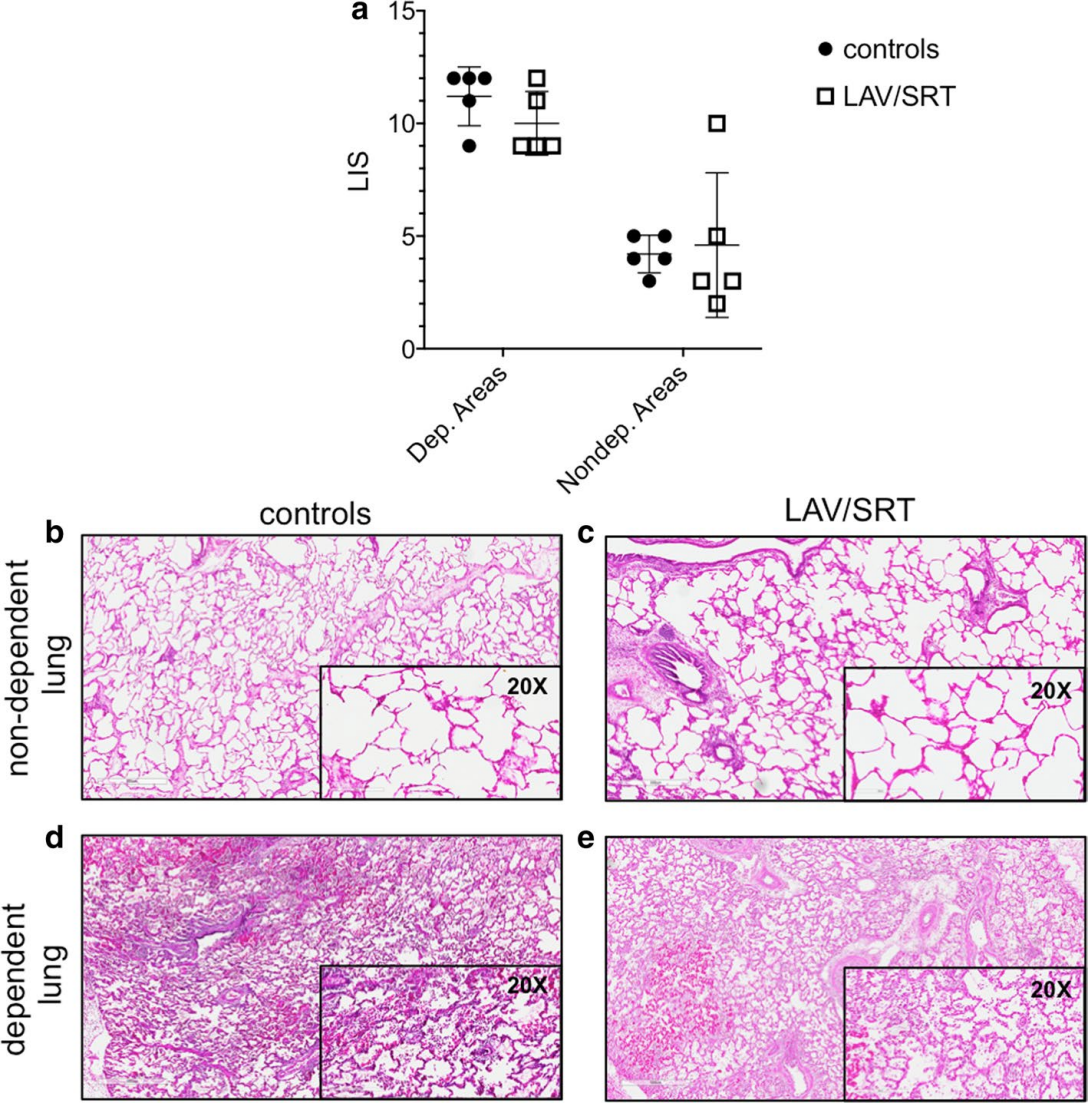
**a** Lung injury score (LIS) of the dependent (dep.) and non-dependent (nondep.) lung tissue, calculated as the average score for each of the following features: infiltrating white blood cells, airspace hemorrhage, vascular congestion, edema, and fibrin deposition. **b**–**e** Representative images (×5 and ×20) of hematoxylin and eosin histologic sections from **b**, **c** non-dependent and **d**, **e** dependent lung tissue biopsies

In both groups, acute lung injury was demonstrated macroscopically (Additional file 1: Figure S3) and in hematoxylin and eosin (HE) histologic sections as evidenced by infiltrating white blood cells, airspace hemorrhage, vascular congestion, edema, and fibrin deposition (Fig. 7). Comparison of HE staining of dependent (posterior lung tissue) and non-dependent (anterior lung tissue) sections of injured lung from the two groups did not reveal a statistically significant difference based on scoring of acute lung injury (*p* = 0.33 and p = 0.66 for dependent and non-dependent, respectively).

## Discussion

A complex, reproducible, clinically relevant, in vivo experimental model of severe ARDS induced by two subsequent bronchoscope instillations of low-pH GJ was established in mechanically ventilated pigs. The criteria for severe ARDS were satisfied in all animals, that is PaO_2_/FiO_2_ ratio < 100 mmHg (Fig. 2), bilateral opacities on CXR (Additional file 1: Figure S1), pulmonary arterial wedge pressure ≤ 18 mmHg (Additional file 1: Table S1) and lastly the acute onset of injury following GJ instillation present in this study. Despite the severity of the lung injury, the support provided by VV-ECMO allowed the maintenance of adequate gas exchange and stable hemodynamic parameters (Fig. 3).

Few other ARDS animal models combined with ECMO support have been described, including injury models with oleic acid infusion, warm saline airway lavage and smoke inhalation [20–22]. However, different from the other published models, our model of severe ARDS is more clinically relevant, as aspiration of gastric contents is a frequent cause of ARDS in clinical practice [1], and causes lung injury with the same mechanisms occurring in the clinical setting. Moreover, ECMO is used as rescue treatment in patients with aspiration ARDS [6], including pregnant women with aspiration pneumonitis after general anesthesia [23]. The consistency and reproducibility of our ARDS model demonstrated by the low variability of the PaO_2_/FiO_2_ ratio post-injury in the two groups is the result of few technical precautions. The GJ was pulled in one container from different donors, the pH was adjusted at 1.6, and the delivery to the airways was provided through bronchoscopic view in order to specifically target each bronchial segment with a specific volume of fluid.

Our consistent and reproducible model permitted the evaluation of one potential therapeutic strategy that included saline lung lavage combined with SRT early in the course of severe ARDS. Bronchoscopy-based treatments would have otherwise not been possible without extracorporeal support in severely hypoxemic subjects. Indeed, the treatment with saline lavage and SRT was physiologically well tolerated by all the animals in the intervention group (Fig. 3), whose gas exchange was maintained in normal range by ECMO. To our knowledge, recent studies on SRT in adult ARDS have not included ECMO patients or included a lung lavage treatment preceding surfactant administration. SRT during ECMO has been studied in pediatric patients and shown to be beneficial [24].

However, the results of our investigation showed that lung physiologic and biologic parameters were not significantly different in treated animals compared with controls (Fig. 3).

A large number of clinical studies focused on the potential therapeutic role of SRT in ARDS, but failed to show a significant effect on mortality [10, 11]. Reasons that may explain the negative results include dosing of surfactant, administration modalities, and lastly the persistent presence in the alveolar space of inflammatory factors, which can cause endogenous and exogenous surfactant dysfunction. Moreover, studies on SRT in adult ARDS have not included severely hypoxemic patients, who may benefit the most from any potential ARDS therapy given the severity of lung injury, but for the same reason would not safely tolerate intra-tracheally delivered therapies.

These issues were addressed in our experimental model. Firstly, we caused severe ARDS requiring VV-ECMO to restore adequate gas exchange and stable physiological conditions to tolerate lung lavage with high volume of saline (10 mL per bronchial segment, for a total of ~ 200 mL, with a return of ~ 100 mL). The ECMO support secondarily allowed comprehensive bronchoscopy in order to remove aspiration contents, inflammatory mediators, and aspiration-induced dysfunctional surfactant, followed by delivery of high doses of exogenous surfactant in each bronchial segment (~ 5 mL (containing 135 mg phospholipid)/kg body weight).

A similar approach was studied by Nakajima and colleagues [16] in a lung transplant-related experimental model to treat mild acid aspiration-induced lung injury (PaO_2_/FiO_2_ ratio 200–300 mmHg) caused in vivo by bronchoscopic instillation of gastric juice. Lungs were treated ex vivo in the EVLP system, which allowed the accurate and safe administration of the therapy independently of gas exchange and the potential associated systemic complications. The results showed that only the combination of lung lavage and SRT, but not lung lavage or SRT alone, resulted in better physiologic lung function and reduced inflammation at the end of EVLP and after lung transplant.

Our study attempted to translate whether this ex vivo approach had broader clinical implications for ARDS treatment, such as in an in vivo setting using VV-ECMO as a platform. Although we employed a similar model of lung injury and a similar therapeutic strategy with lung lavage and SRT, several features in our model may explain the different results from Nakajima and colleagues work. First, the severity of lung injury was considerably higher in our model, as only mild ARDS was achieved in Nakajima and colleagues based on PaO_2_/FiO_2_. The more severe lung consolidation in our model may have prevented the exogenous surfactant to adequately reach the alveolar space. Second, the absence of chest wall in the EVLP system may have facilitated lung recruitment with consequent higher exogenous surfactant bioavailability in the alveolar space. Indeed, SRT in combination with lung RM has been shown effective to improve oxygenation and lung volume [24–26]. It would be hence interesting to investigate whether SRT is more effective in ARDS subjects with higher alveolar ‘recruitability’ compared to subjects with persistent lung consolidation. Third, due to the severity and extension of lung injury in our model, the dose of exogenous surfactant may have been insufficient, or the lung lavage may have not been as efficient to remove aspiration contents and the products of the consequent pulmonary inflammatory response. Indeed, in our model the total BA concentrations from BAL, although lower in the treatment group (Fig. 6), were not found to be significantly different from controls. Perhaps performing the lavage with surfactant itself, as suggested by the results in a lung contusion model of ARDS [17], could take advantage of its adsorption properties and facilitate distribution and subsequent recovery. However, even exogenous surfactant could have been degraded by the activity of specific enzymes, including the secretory phospholipase A2 [27], which in patients with direct forms of ARDS has been shown to inversely correlate with PaO_2_/FiO_2_ ratio and mortality [28]. Alternatively, it is possible that lung lavage itself had worsened the injury in the peripheral, ventilated alveolar units, increasing lung consolidation and preventing alveolar delivery of surfactant, or increasing the air–water surface tension, which is recognized as one of the mechanisms of cellular damage and lung injury propagation [29]. By these mechanisms, lung lavage may have also caused an injurious response to the recruitment maneuver only in the intervention group. Finally, while in our model lungs were physiologically perfused with blood, which may sustain the inflammatory response to the acute insult in the lung, in the EVLP system lungs are perfused with an acellular solution, which may blunt inflammation and facilitate lung healing.

Our study has a number of limitations. The complexity of the model and the amount of resources required to perform the experiments restricted the number of animals included in each experimental group. A dose response evaluation with different amount of saline for lung lavage and increasing doses of surfactant for the SRT was not performed. Thus, an optimal dose for efficacy in this model was not determined. Although our rationale for our dosage stemmed from computational data by Filoche and colleagues [30], the work of Nakajima et al. [16] and the manufacturer recommendation, it may have been inadequate in our experimental model. Our experimental design and timing may have also influenced the observed results. The duration after lavage and SRT that the animal was monitored was relatively short and thus may have precluded the possibility of observing a beneficial effect from the therapy. Our protocol monitored the animal for 5 h after therapy (4 h on ECMO/1 h off). Previous surfactant studies, instead, monitored subjects for extended periods, often past 4 h after surfactant was administered [13, 14, 17, 18, 25, 26]. Furthermore, in studies where bronchoscopically administered surfactant did show improvements in oxygenation, benefits were observed > 24 h after treatment [13, 18, 26]. Thus, longer follow-up after SRT during ECMO will need to be investigated in future studies.

Further investigations should also address whether different timing and doses of the treatment strategy, including treatment with surfactant replacement only, may be effective in reducing injury and facilitate lung healing. Moreover, the effect of different mechanical ventilation strategies, resulting in better alveolar recruitment, could potentially improve the distribution of the surfactant to the injured areas of the lung. Alternatively, it is possible that the treatment with saline lavage and surfactant replacement is not efficacious in this aspiration model of severe ARDS.

## Conclusions

In conclusion, a reproducible pre-clinical model of aspiration-induced severe ARDS requiring VV-ECMO was successfully established. Despite the severity of lung injury, VV-ECMO support allowed the maintenance of adequate gas exchange and stable hemodynamic parameters, which allowed investigation of the efficacy of a therapeutic strategy consisting of lung lavage and SRT. The treatment resulted in a transient decrease in lung compliance and oxygenation immediately post-therapy, but was overall well tolerated. However, at the end of each experiment, the lung function parameters—PaO_2_/FiO_2_, pCO_2_, respiratory rate and compliance—in the treatment group were not different than controls.

## Supplementary information

**Additional file 1:** **Table S1.** Baseline, experimental and intraoperative variables between CONTROL and LAV/SRT groups. **Figure S1.** A baseline preinjury chest xray is shown above on the left and a subsequent xray 2 hours after the GJ was instilled. The chest xray on the right demonstrates bilateral opacities typical of ARDS. The pulmonary artery catheter, esophageal temperature probe, ECMO drainage and return cannulas can also be seen on both images. **Figure S2.** Representative bronchoscopy images during course of experiment. **(A)** shows the preinjury airways **(B)** taken during instillation of gastric juice **(C)** shows the airways 2 hours after the injury and **(D)** shows the instillation of surfactant in the LAV/SRT group. **Figure S3.** Representative gross image of lungs after median sternotomy at the conclusion of one of the cases. Two consecutive instillations of gastric juice consistently reproduced the bilateral, dishomogeneous distribution of injury observed above. The most injured areas of the lung were often at the posterior bases in keeping with the animal’s supine position during the experiment. The impression of the PA catheter within the right ventricle can be appreciated in the heart and the tip of the ECMO drainage cannula in the inferior vena cava is observed at the bottom right of the photograph.

## Data Availability

All data generated or analyzed during this study are included in this published article and its additional file.

## Abbreviations

ARDS: Acute respiratory distress syndrome
VV-ECMO: Veno-venous extracorporeal membrane oxygenation
SRT: Surfactant replacement therapy
EVLP: Ex vivo lung perfusion
PCV: Pressure control ventilation
VCV: Volume controlled ventilation
PEEP: Positive end expiratory pressure
FiO_2_: Fraction of inspired oxygen
RR: Respiratory rate
PA: Pulmonary artery
GJ: Gastric juice
LAV: Lavage
RM: Recruitment maneuver
BA: Bile acid
BAL: Bronchoalveolar lavage fluid
MAP: Mean arterial pressure
HR: Heart rate
SD: Standard deviation
HE: Hematoxylin and eosin

## References

[1] Bellani G, Laffey JG, Pham T, Fan E, Brochard L, Esteban A, Gattinoni L, van Haren F, Larsson A, McAuley DF, Ranieri M, Rubenfeld G, Thompson BT, Wrigge H, Slutsky AS, Pesenti A, Investigators LS, Group ET (2016). Epidemiology, patterns of care, and mortality for patients with acute respiratory distress syndrome in intensive care units in 50 countries. JAMA.

[2] Force ADT, Ranieri VM, Rubenfeld GD, Thompson BT, Ferguson ND, Caldwell E, Fan E, Camporota L, Slutsky AS (2012). Acute respiratory distress syndrome: the Berlin definition. JAMA.

[3] Duggal A, Ganapathy A, Ratnapalan M, Adhikari NK (2015). Pharmacological treatments for acute respiratory distress syndrome: systematic review. Minerva Anestesiol.

[4] Gallo de Moraes A, El-Yafawi R, Oeckler RA (2019). Early Neuromuscular blockade in the acute respiratory distress syndrome. N Engl J Med.

[5] Fan E, Del Sorbo L, Goligher EC, Hodgson CL, Munshi L, Walkey AJ, Adhikari NKJ, Amato MBP, Branson R, Brower RG, Ferguson ND, Gajic O, Gattinoni L, Hess D, Mancebo J, Meade MO, McAuley DF, Pesenti A, Ranieri VM, Rubenfeld GD, Rubin E, Seckel M, Slutsky AS, Talmor D, Thompson BT, Wunsch H, Uleryk E, Brozek J, Brochard LJ, American Thoracic Society ESoICM, Society of Critical Care M (2017). An Official American Thoracic Society/European Society of Intensive Care Medicine/Society of Critical Care Medicine Clinical Practice Guideline: Mechanical Ventilation in Adult Patients with Acute Respiratory Distress Syndrome. Am J Respir Crit Care Med.

[6] Brodie D, Slutsky AS, Combes A (2019). Extracorporeal life support for adults with respiratory failure and related indications: a review. JAMA.

[7] Del Sorbo L, Cypel M, Fan E (2014). Extracorporeal life support for adults with severe acute respiratory failure. Lancet Respir Med.

[8] Raghavendran K, Willson D, Notter RH (2011). Surfactant therapy for acute lung injury and acute respiratory distress syndrome. Crit Care Clin.

[9] Willson DF, Notter RH (2011). The future of exogenous surfactant therapy. Respir Care.

[10] Baudouin SV (2004). Exogenous surfactant replacement in ARDS–one day, someday, or never?. N Engl J Med.

[11] Dushianthan A, Cusack R, Goss V, Postle AD, Grocott MP (2012). Clinical review: exogenous surfactant therapy for acute lung injury/acute respiratory distress syndrome–where do we go from here?. Crit Care.

[12] Willson DF, Truwit JD, Conaway MR, Traul CS, Egan EE (2015). The adult calfactant in acute respiratory distress syndrome trial. Chest.

[13] Amital A, Shitrit D, Raviv Y, Saute M, Medalion B, Bakal L, Kramer MR (2008). The use of surfactant in lung transplantation. Transplantation.

[14] Gunther A, Schmidt R, Harodt J, Schmehl T, Walmrath D, Ruppert C, Grimminger F, Seeger W (2002). Bronchoscopic administration of bovine natural surfactant in ARDS and septic shock: impact on biophysical and biochemical surfactant properties. Eur Respir J.

[15] Lewis J, McCaig L, Hafner D, Spragg R, Veldhuizen R, Kerr C (1999). Dosing and delivery of a recombinant surfactant in lung-injured adult sheep. Am J Respir Crit Care Med.

[16] Nakajima D, Liu M, Ohsumi A, Kalaf R, Iskender I, Hsin M, Kanou T, Chen M, Baer B, Coutinho R, Maahs L, Behrens P, Azad S, Martinu T, Waddell TK, Lewis JF, Post M, Veldhuizen RAW, Cypel M, Keshavjee S (2017). Lung lavage and surfactant replacement during ex vivo lung perfusion for treatment of gastric acid aspiration-induced donor lung injury. J Heart Lung Transplant.

[17] Strohmaier W, Trupka A, Pfeiler C, Thurnher M, Khakpour Z, Gippner-Steppert C, Jochum M, Redl H (2005). Bilateral lavage with diluted surfactant improves lung function after unilateral lung contusion in pigs. Crit Care Med.

[18] Walmrath D, Grimminger F, Pappert D, Knothe C, Obertacke U, Benzing A, Gunther A, Schmehl T, Leuchte H, Seeger W (2002). Bronchoscopic administration of bovine natural surfactant in ARDS and septic shock: impact on gas exchange and haemodynamics. Eur Respir J.

[19] Meers CM, De Wever W, Verbeken E, Mertens V, Wauters S, De Vleeschauwer SI, Vos R, Vanaudenaerde BM, Verleden GM, Van Raemdonck DE (2011). A porcine model of acute lung injury by instillation of gastric fluid. J Surg Res.

[20] Araos J, Alegria L, Garcia P, Damiani F, Tapia P, Soto D, Salomon T, Rodriguez F, Amthauer M, Erranz B, Castro G, Carreno P, Medina T, Retamal J, Cruces P, Bugedo G, Bruhn A (2016). Extracorporeal membrane oxygenation improves survival in a novel 24-hour pig model of severe acute respiratory distress syndrome. Am J Transl Res.

[21] Langer T, Vecchi V, Belenkiy SM, Cannon JW, Chung KK, Cancio LC, Gattinoni L, Batchinsky AI (2014). Extracorporeal gas exchange and spontaneous breathing for the treatment of acute respiratory distress syndrome: an alternative to mechanical ventilation?*. Crit Care Med.

[22] Shekar K, Fung YL, Diab S, Mullany DV, McDonald CI, Dunster KR, Fisquet S, Platts DG, Stewart D, Wallis SC, Smith MT, Roberts JA, Fraser JF (2012). Development of simulated and ovine models of extracorporeal life support to improve understanding of circuit-host interactions. Crit Care Resusc.

[23] Ong J, Zhang JJY, Lorusso R, MacLaren G, Ramanathan K (2020). Extracorporeal membrane oxygenation in pregnancy and the postpartum period: a systematic review of case reports. Int J Obstet Anesth.

[24] Hermon M, Burda G, Male C, Boigner H, Ponhold W, Khoss A, Strohmaier W, Trittenwein G (2005). Surfactant application during extracorporeal membrane oxygenation improves lung volume and pulmonary mechanics in children with respiratory failure. Crit Care.

[25] Lu Q, Zhang M, Girardi C, Bouhemad B, Kesecioglu J, Rouby JJ (2010). Computed tomography assessment of exogenous surfactant-induced lung reaeration in patients with acute lung injury. Crit Care.

[26] Tsangaris I, Galiatsou E, Kostanti E, Nakos G (2007). The effect of exogenous surfactant in patients with lung contusions and acute lung injury. Intensive Care Med.

[27] Seeds MC, Grier BL, Suckling BN, Safta AM, Long DL, Waite BM, Morris PE, Hite RD (2012). Secretory phospholipase A2-mediated depletion of phosphatidylglycerol in early acute respiratory distress syndrome. Am J Med Sci.

[28] Nakos G, Kitsiouli E, Hatzidaki E, Koulouras V, Touqui L, Lekka ME (2005). Phospholipases A2 and platelet-activating-factor acetylhydrolase in patients with acute respiratory distress syndrome. Crit Care Med.

[29] Cressoni M, Chiumello D, Algieri I, Brioni M, Chiurazzi C, Colombo A, Colombo A, Crimella F, Guanziroli M, Tomic I, Tonetti T, Luca Vergani G, Carlesso E, Gasparovic V, Gattinoni L (2017). Opening pressures and atelectrauma in acute respiratory distress syndrome. Intensive Care Med.

[30] Filoche M, Tai CF, Grotberg JB (2015). Three-dimensional model of surfactant replacement therapy. Proc Natl Acad Sci USA.

