# Supplementary material for: Veno-venous ECMO as a platform to evaluate lung lavage and surfactant replacement therapy in an animal model of severe ARDS
Source: Intensive Care Med Exp. 2020 Oct 27;8:63. doi: 10.1186/s40635-020-00352-w (PMC7591687; doi:10.1186/s40635-020-00352-w)

VV-ECMO as a platform to evaluate bronchoscopic saline lavage and surfactant therapy in severe ARDS

Robert Qaqish^1^ MD MSc, Yui Watanabe^1^ MD PhD, Marcos Galasso^1^ MD, Cara Summers^1^, Aadil Ali^1^, Mamoru Takahashi^1^ MD, Anajara Gazzalle^1^ MD, Sassan Azad^1^, Mingyao Liu^1^ MD MSc, Shaf Keshavjee^1^ MD MSc, Marcelo Cypel^1^ MD MSc, and Lorenzo Del Sorbo^1,2^ MD

**Additional file**

**DETAILED MATERIALS AND METHODS**

The animals in our study were treated in accordance with the guidelines outlined in the ‘Guide for the Care and use of Laboratory Animals’, published by the National Research Council. Our animal use protocol was approved by the Toronto General Research Institute Animal Care Committee. Yorkshire male domestic pigs (29-37 kg) were used for this study. Pigs were induced using ketamine (20mg/kg intramuscular [IM]), midazolam (0.3 mg/kg IM) and atropine (0.04 mg/kg IM). General anesthesia was maintained using a propofol infusion (12-20 mg/kg) and inhaled isoflurane (4%) and analgesia was provided with remifentanil (19-30 ug/kg). Augmentation of the anesthesia was sometimes required to ensure that the set respiratory rate matched the animal’s respiratory rate and that triggering of the ventilator was minimized since paralysis was not used. Each of the animals were maintained with a 0.9% NaCl (Baxter Corp, Mississauga, ON) infusion via central venous access at 5-10 ml/kg. A 38 degree heating blanket maintained normothermia and an esophageal temperature probe measured core body temperature.

Animals underwent open tracheostomy and a 7.5 mm I.D (Mallinckrodt, Covidien, Mansfield, MA) endotracheal tube (ETT) was used. The left internal carotid artery was cannulated and secured using an 8 French (Fr) pediatric feeding tube (Covidien, Mansfield, MA) for serial blood pressure measurement and arterial blood gas analysis. Blood gas analysis was conducted immediately post tracheostomy and every 15-60 minutes depending on the event during the experiment using a Siemens blood gas analyzer (Rapidpoint 500, Seimens, Malvern, PA). After the airway was secured and arterial line placed, a 10 cm, 8.5 Fr percutaneous introducer (Arrow/Teleflex, Morrisville, NC) was sterilely placed in the left external jugular (EJ) vein under ultrasound guidance. A 7.5 Fr pulmonary artery catheter (Edwards Life Sciences, Irvine CA) was subsequently placed via pressure wave guidance from the left EJ. Heart rate (beats per min), respiratory rate (breaths per minute, bpm), end-tidal carbon dioxide (etCO_2_), arterial blood pressure (mmHg), pulmonary artery (PA) pressure, esophageal temperature, and continuous pulse oximetry were used to continuously monitor the animal and were recorded. Pulmonary capillary wedge pressure (PCWP) was also measured.

**Ventilator Management:** The animals were ventilated (Servo-i, Maquet Getinge Group, Wayne, NJ) using pressure control ventilation (PCV) for the majority of the experiment. A mechanical filter intervened between the ventilator tubing and ETT (Covidien, Mansfield, MA). Alarms were adjusted on the ventilator such that the upper limit of the respiratory rate and peak airway pressures were 50 bpm and 60 cmH_2_O, respectively. Prior to injury with gastric juice, PCV was set with a driving pressure of 15 cmH_2_O and peak end-expiratory pressure (PEEP) of 5 cmH_2_O (PCV 15/5), fraction of inspired oxygen (FiO_2_) of 50%, respiratory rate (RR) of 15-20, breaths per minute (bpm), inspiratory to expiratory ratio (I:E) 1:2 seconds, 5% inspiratory rise and -5 cmH_2_O trigger pressure. For 5 minutes immediately after each instillation of gastric juice contents, the mode was changed to volume controlled ventilation (VCV) 10 mL/kg, 100% FiO_2_ and the respiratory rate adjusted immediately post injury in response to hypercapnia. After 5 minutes of VCV at 10 mL/kg the settings were changed back to the original pressure controlled settings with the FiO_2_ kept at 100%.

The rationale for this change in ventilation mode post injury stemmed from results from our pilot experiments. As a result of a large volume of gastric juice being instilled in the airways, the airway resistance increases dramatically and almost obstructs the lumen of the airway. As a result, severe hypercapnia ensues secondary to low tidal volume ventilation on low pressure control ventilation (PCV 15/5). Volume control settings guaranteed sufficient tidal ventilation immediately post injury and further minimized dangerous levels of CO_2_. Furthermore, VCV was also used to help distribute the gastric juice more peripherally.

After ECMO was initiated and the animal started to show improvement in oxygen saturation/etCO_2_, the FiO_2_ was decreased to 50% for the remainder of the experiment. This was completed within the first 5 minutes after ECMO initiation.

In both controls and treatment cases, recruitment maneuvers were used. In controls, at 2 hours post injury while on ECMO, a recruitment maneuver included 3 sustained inflations, 10 seconds each with airway pressure of 30 cmH_2_O, during a 5 minute period of VCV at 10 ml/kg. PCV 15/5 was resumed after this 5 minute period. For treatment groups, PCV 15/5 was used during the saline lavage and VCV 10 mL/kg was used during surfactant instillation. 3 sustained inflations, 10 seconds each at airway pressure of 30 cmH_2_O were delivered after the active administration of the surfactant.

**ECMO Cannulation and management:** After the pulmonary artery catheter was placed, the right neck and groin were prepared and draped in a sterile fashion. Ultrasound was used prior to cutting down the right neck to plan our skin incision. A 15 Fr (5.0 mm x 50 cm, Bio-medicus venous cannula, Medtronic, Minneapolis, MN) was used for the femoral vein (drainage cannula) and a 14 Fr (4.7 mm x 10 cm, Bio-medicus arterial cannula, Medtronic, Minneapolis, MN) was used for the external jugular vein (return cannula). Chest radiography was used to confirm cannula positioning. Just prior to cannulation, systemic heparinization was achieved with 5000 IU of heparin (Heparin LEO, LEO Pharma Inc, Thornhill, ON, Canada) and subsequently, 1000 IU were administered every hour for the remainder of the experiment. The circuit consisted of a Quadrox-i Adult microporous membrane oxygenator (HMO 70000, Maquet Getinge Group, Wayne, NJ), pump pack custom perfusion system (CB7D91R9, Medtronic Minneapolis, MN), centrifugal pump (050300000, Sorin, Mirandola, Italy), pump head (Stockert, Sorin Group, Arvada, CO) and heat exchanger (Terumo Cardiovascular Group, Ann Arbor, MI). All animals received a fluid bolus of 200 mL just prior to ECMO initiation. Maximum flow (L/min) was guided by animal hemodynamics such that goal flow rate was maximized when MAP fell to 65 mmHg. Sweep was turned on (2 L/min) one hour after the first injury was administered.

**Injury with gastric juice:** Gastric juice (GJ) was collected from pigs of similar weight and pooled. The GJ was initially filtered through surgical gauze and the pH was measured and titrated to a pH of approximately 1.6 and stored at -80 degrees Celsius. On the day of the experiment the pH was measured again to ensure stability of pH and kept in a 37 degree Celsius water bath until required for injury.

Instillation of gastric juice was completed using a bronchoscope (Olympus BF Type 160, Exera II CLV-180/CV180, Tokyo, Japan). The first instillation of gastric injury occurred using a volume of 4 ml/kg. The cranial lobes (L and R) received 25% (12.5% each), right accessory lobe (12.5%), right middle lobe (12.5%), right and left lower lobes received 50%. In each segment the bronchoscope was placed as peripherally as possible and the gastric juice was instilled slowly under direct visualization of the airway. After completion of the first injury, the ventilation was switched to VCV 10 mL/kg, FiO_2_ 100%. Thirty minutes after this first injury, a second injury was given (2 mL/kg, 50% in each lower lobes) followed by 5 minutes of VCV (10 mL/kg, FiO_2_ 100%). Serial blood gas analysis occurred every 15 minutes for a one hour period before which the sweep gas (2 L/min) was turned on.

**Saline lavage and surfactant administration:** Two hours after the first injury in our treatment group, 200 mL of (0.9% NaCl) saline was used to lavage the lungs. Approximately 20 segments were lavaged with 10 mL per segment. Fluid recovered from segments was intermittently collected into a specimen trap (Argyle, Covidien, Mansfield, MA) attached to the suction valve port and the total volume recorded.

Surfactant (BLES Pharmaceuticals, London, Ontario, Canada) was used in our treatment group. Dosing for our treatment group was based on the manufacturer recommended dose for neonates with respiratory distress syndrome (135 mg/kg, 27 mg/mL). After the 200 mL saline lavage, surfactant was instilled in approximately 20 segments. Each segmental administration was followed with a 5 mL air bolus to clear the channel of any surfactant fluid remaining. Before each administration of the surfactant bolus, inspiration was held to maximize the chances that the airways were open prior to surfactant instillation. After surfactant instillation, ventilation was switched to VCV 10 mL/kg for 5 minutes and 3 sustained inflations (10 sec) were performed.

**Plasma collection and BAL collection :** Bronchial alveolar lavage (BAL) was performed immediately before injury, 2 hours after the first injury and 4 hours after either the recruitment maneuver only (controls) and lavage/surfactant/recruitment (treatment). Two 25 mL aliquots of 0.9% NaCl (Baxter, Mississauga, ON) were instilled in the right middle lobe of the animal during these times. Each BAL was centrifuged (Centrifuge 5810R 15A, Eppendorf AG, Mississauga, ON) at 4 degrees for 10 minutes at 150g. The supernatant was collected and stored at -80 degrees Celsius until analysis was completed.

Blood was collected at the same time points that the BAL was collected, described above. Blood was centrifuged (Centrifuge 5810R 15A, Eppendorf AG, Mississauga, ON) at 4 degrees for 10 minutes at 150g. The supernatant was collected and stored at -80 degrees Celsius until analysis was completed.

**Table S1**: Baseline, experimental and intraoperative variables between CONTROL and LAV/SRT groups.

|  | **CONTROL (n=5)** | **LAV/SRT (n=5)** |  |
| --- | --- | --- | --- |
|  | mean (SD) | mean (SD) | p-value |
| Animal weight (kg) | 32.2 (1.7) | 33.02 (1.85) | 0.421 |
| pH of gastric juice | 1.684 (0.07) | 1.654 (0.06) | 0.674 |
| Volume of gastric juice/body weight (mL/kg) | 5.97 (0.14) | 6.13 (0.3) | 0.889 |
| Hemoglobin (g/L) (baseline) | 102.8 (4.02) | 110.2 (5.81) | 0.087 |
| Estimated blood loss (mL) | 12 (7.58) | 11 (4.18) | >0.999 |
| Blood flow on ECMO* (L/min) | 1.254 (0.22) | 1.57 (0.22) | 0.095 |
| partial pressure of CO_2_ (pCO_2_ mmHg) | 43 (1) | 44 (1) | 0.34 |
| Expired tidal volume (mL) | 374 (6) | 354 (33) | 0.246 |
| Respiratory rate (breaths per min) | 15 (1) | 17 (2) | 0.08 |
| pH | 7.43 (0.03) | 7.41 (0.01) | 0.54 |
| Time on ventilator prior to injury (min) | 219.4 (41.3) | 203.6 (24.3) | 0.937 |
| PaO_2_/FiO_2_ ratio (mmHg) | 485 (15) | 490 (25) | >0.999 |
| Preinjury PCWPꝉ (mmHg) | 7.2 (4.32) | 7.8 (4.81) | 0.999 |
| Total intravenous fluid administration (mL) | 2554.8 (929) | 2471.2 (333) | 0.999 |
| BAL‡ bile acid concentration (µmol/L) | 0.67 (1.38) | 0.03 (0.02) | 0.175 |
| Recovered saline lavage (200 mL) | n/a | 134.3 (16.6) | n/a |
| Volume of surfactant used (mL) | n/a | 164.8 (9.09) | n/a |
| *sweep = 2 L/min for both groups; ꝉpulmonary capillary wedge pressure; ‡bronchoalveolar lavage (preinjury); p-value calculated by Mann-Whitney test | | | |

**Figure legend.**

Figure S1: A baseline preinjury chest xray is shown above on the left and a subsequent xray 2 hours after the GJ was instilled. The chest xray on the right demonstrates bilateral opacities typical of ARDS. The pulmonary artery catheter, esophageal temperature probe, ECMO drainage and return cannulas can also be seen on both images.


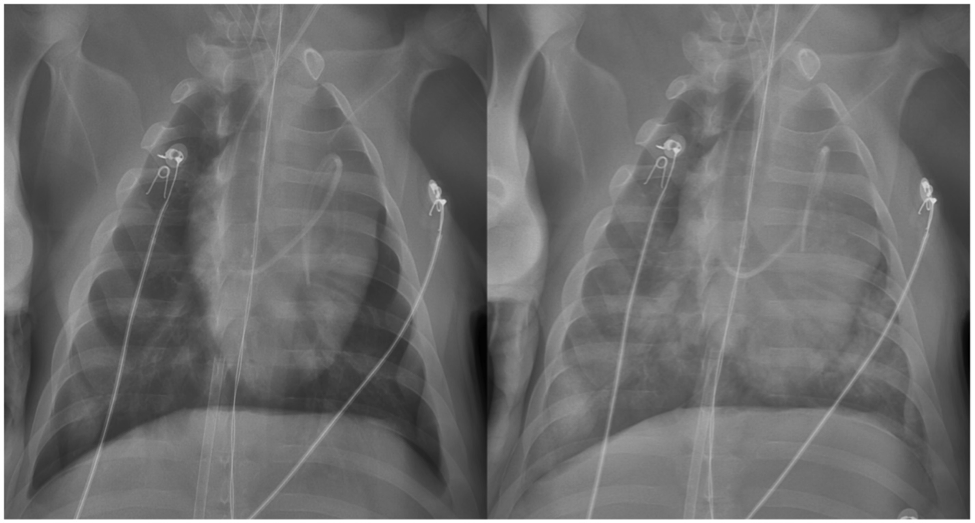


Figure S2: Representative bronchoscopy images during course of experiment. **(A)** shows the preinjury airways **(B)** taken during instillation of gastric juice **(C)** shows the airways 2 hours after the injury and **(D)** shows the instillation of surfactant in the LAV/SRT group.


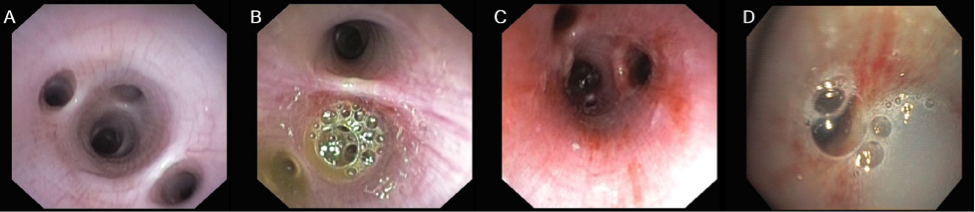


Figure S3: representative gross image of lungs after median sternotomy at the conclusion of one of the cases. Two consecutive instillations of gastric juice consistently reproduced the bilateral, dishomogenous distribution of injury observed above. The most injured areas of the lung were often at the posterior bases in keeping with the animal’s supine position during the experiment. The impression of the PA catheter within the right ventricle can be appreciated in the heart and the tip of the ECMO drainage cannula in the inferior vena cava is observed at the bottom right of the photograph.


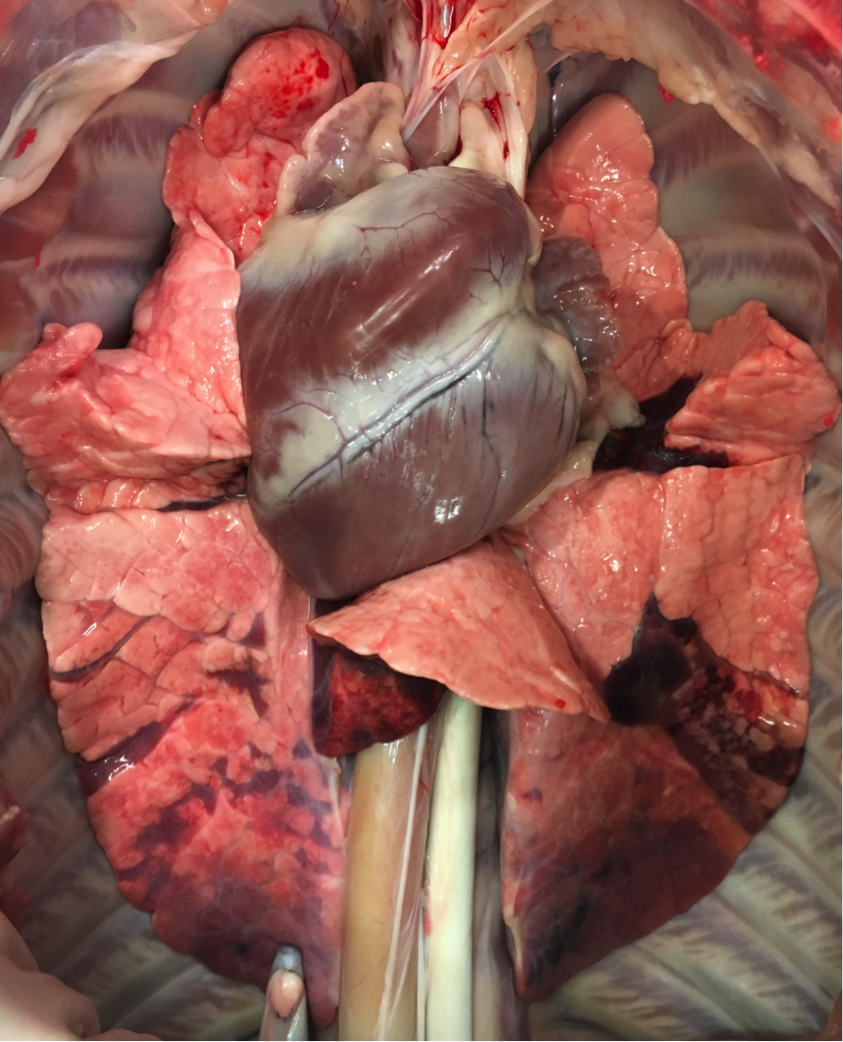

Supplement: Supplementary file 1 — Additional file 1: Table S1. Baseline, experimental and intraoperative variables between CONTROL and LAV/SRT groups. Figure S1. A baseline preinjury chest xray is shown above on the left and a subsequent xray 2 hours after the GJ was instilled. The chest xray on the right demonstrates bilateral opacities typical of ARDS. The pulmonary artery catheter, esophageal temperature probe, ECMO drainage and return cannulas can also be seen on both images. Figure S2. Representative bronchoscopy images during course of experiment. (A) shows the preinjury airways (B) taken during instillation of gastric juice (C) shows the airways 2 hours after the injury and (D) shows the instillation of surfactant in the LAV/SRT group. Figure S3. Representative gross image of lungs after median sternotomy at the conclusion of one of the cases. Two consecutive instillations of gastric juice consistently reproduced the bilateral, dishomogeneous distribution of injury observed above. The most injured areas of the lung were often at the posterior bases in keeping with the animal’s supine position during the experiment. The impression of the PA catheter within the right ventricle can be appreciated in the heart and the tip of the ECMO drainage cannula in the inferior vena cava is observed at the bottom right of the photograph. [file 40635_2020_352_MOESM1_ESM.docx]
